# Consumption and Metabolism of Extracellular Pyruvate by Cultured Rat Brain Astrocytes

**DOI:** 10.1007/s11064-022-03831-6

**Published:** 2022-12-10

**Authors:** Nadine Denker, Antonia R. Harders, Christian Arend, Ralf Dringen

**Affiliations:** Centre for Biomolecular Interactions Bremen, Faculty 2 (Biology/Chemistry) and Centre for Environmental Research and Sustainable Technologies, University of Bremen, P.O. Box 330440, 28334 Bremen, Germany

**Keywords:** Astrocytes, Metabolism, MCT1, Mitochondria, Pyruvate carrier, Transport

## Abstract

Brain astrocytes are considered as glycolytic cell type, but these cells also produce ATP via mitochondrial oxidative phosphorylation. Exposure of cultured primary astrocytes in a glucose-free medium to extracellular substrates that are known to be metabolised by mitochondrial pathways, including pyruvate, lactate, beta-hydroxybutyrate, alanine and acetate, revealed that among the substrates investigated extracellular pyruvate was most efficiently consumed by astrocytes. Extracellular pyruvate was consumed by the cells almost proportional to time over hours in a concentration-dependent manner with apparent Michaelis–Menten kinetics [K_m_ = 0.6 ± 0.1 mM, V_max_ = 5.1 ± 0.8 nmol/(min × mg protein)]. The astrocytic consumption of pyruvate was strongly impaired in the presence of the monocarboxylate transporter 1 (MCT1) inhibitor AR-C155858 or by application of a 10-times excess of the MCT1 substrates lactate or beta-hydroxybutyrate. Pyruvate consumption by viable astrocytes was inhibited in the presence of UK5099, an inhibitor of the mitochondrial pyruvate carrier, or after application of the respiratory chain inhibitor antimycin A. In contrast, the mitochondrial uncoupler BAM15 strongly accelerated cellular pyruvate consumption. Lactate and alanine accounted after 3 h of incubation with pyruvate for around 60% and 10%, respectively, of the pyruvate consumed by the cells. These results demonstrate that consumption of extracellular pyruvate by astrocytes involves uptake via MCT1 and that the velocity of pyruvate consumption is strongly modified by substances that affect the entry of pyruvate into mitochondria or the activity of mitochondrial respiration.

## Introduction

In brain, astrocytes almost completely cover the brain capillaries with their endfeet [1] and thereby have a highly important strategic position to control and regulate the transport of substances from the blood into the brain and vice versa. Furthermore, astrocytes are strongly involved in brain homeostasis and metabolism as essential partners of neurons [2, 3]. Concerning their metabolism, astrocytes are considered as rather glycolytic cell type, but astrocytes do also efficiently use their mitochondrial pathways for ATP generation and many other important metabolic processes [2, 4–7].

Glucose is the predominant substrate of astrocytic metabolism and serves as main carbon and energy source [2, 8, 9]. However, astrocytes at least in culture are also able to take-up and metabolize other substrates for ATP production, including other sugars such as mannose and fructose [10, 11], ketone bodies [12, 13], acetate [14] and amino acids [15–17].

The glucose taken up by astrocytes is mainly metabolized via glycolysis [2]. End product of aerobic glycolysis is pyruvate, a 3-carbon α-keto acid that is a highly important metabolic intermediate which serves as substrate for various enzymes and metabolic pathways. For astrocytes it is known that intracellular pyruvate can be efficiently reduced to lactate by lactate dehydrogenase [18, 19], oxidatively decarboxylated by mitochondrial pyruvate dehydrogenase to acetyl-CoA [7, 20] or carboxylated by mitochondrial pyruvate carboxylase to oxaloacetate [6, 7]. In addition, pyruvate can be transaminated to alanine by glutamate-pyruvate transaminases as astrocytes express both the cytosolic GPT1 and mitochondrial GPT2 isoenzymes [21].

For mitochondrial metabolism of pyruvate, cytosolic pyruvate has to be imported into mitochondria. This transport is mediated by the mitochondrial pyruvate carrier (MPC), a hetero-oligomeric complex formed by the two proteins MPC1 and MPC2 [22, 23]. MPC is functionally expressed in astrocytes [23]. Within the mitochondrial matrix of astrocytes, the pyruvate dehydrogenase complex (PDC) oxidatively decarboxylates pyruvate to acetyl coenzyme A (acetyl-CoA) [7, 20]. The activity of this enzyme has been reported to limit the oxidative metabolism of pyruvate and of substances which are metabolized via pyruvate [24]. Astrocytes in culture have been reported to maintain a rather low activity of PDC under unstressed conditions [24]. Acetyl-CoA can enter the TCA cycle in astrocytes [8] or can be used for the synthesis of the ketone bodies acetoacetate and beta-hydroxybutyrate (bHB) [25, 26]. The oxaloacetate needed for citrate synthesis with acetyl CoA can be generated by the anaplerotic pyruvate carboxylase which is considered as an astrocyte specific enzyme in brain [27, 28].

Astrocytes have been reported to release pyruvate and such astrocyte-derived extracellular pyruvate has been shown to be antioxidative and neuroprotective [29, 30]. Extracellular pyruvate can also be taken up by astrocytes in a saturable and pH-dependent process [31], mainly mediated by the monocarboxylate transporter (MCT) 1 which is expressed in astrocytes [32–34] and has a K_m_ value of around 1 mM for its substrate pyruvate [35]. Studies using radioactively labelled pyruvate revealed that pyruvate is efficiently taken up and rapidly converted to lactate and alanine in cultured astrocytes [31].

Despite the importance of pyruvate as central cellular metabolic intermediate, surprisingly little information is available on the processes which are involved in the consumption of extracellular pyruvate by astrocytes. To address such questions, we have investigated the transport processes involved in astrocytic pyruvate consumption as well as mitochondrial processes which may modulate astrocytic pyruvate consumption. The results presented here demonstrate that, compared to other substrates of mitochondrial metabolism, astrocytes consume pyruvate more efficiently in the absence of glucose and that MCT1 as well as the mitochondrial pyruvate carrier are involved in the consumption of extracellular pyruvate. Furthermore, pyruvate consumption by astrocytes depends strongly on the activity of mitochondrial respiration. Finally, the main products of pyruvate consumption by glucose-starved astrocytes are lactate and alanine, which are found to be released from these cells.

## Materials and Methods

### Materials

Dulbecco’s modified Eagles medium (DMEM) and penicillin G/streptomycin sulfate solution (Pen/Strep) were obtained from Thermo Fisher Scientific (Schwerte, Germany). Fetal calf serum (FCS), antimycin A, BAM15 and UK5099 were purchased from Sigma-Aldrich (Darmstadt, Germany). AR-C155858 was purchased at Tocris (Bristol, UK). All enzymes used were purchased from Roche Diagnostics (Mannheim, Germany). The Acetic Acid Assay kit (ACS Manual Format) was from Megazyme (Bray, Ireland). The Cell Titer Glo® 2.0 Assay Kit for ATP quantification was from Promega (Walldorf, Germany). Other chemicals of the highest purity available were obtained from Merck (Darmstadt, Germany), Sigma-Aldrich (Darmstadt, Germany), Roth (Karlsruhe, Germany), AppliChem (Darmstadt, Germany) or Thermo Fisher Scientific (Schwerte, Germany). Sterile cell culture materials and unsterile 96-well plates and black microtiter plates were purchased from Sarstedt (Nümbrecht, Germany).

### Astrocyte Cultures

Astrocyte-rich primary cultures were prepared as previously described in detail from the brains of newborn Wistar rats [36]. Cells were seeded in a density of 300,000 cells per well in 1 mL culture medium (90% DMEM containing 25 mM glucose, 44.6 mM sodium bicarbonate, 1 mM pyruvate, 20 U/mL penicillin G, 20 µg/mL streptomycin sulfate, supplemented with 10% FCS) into wells of 24-well dishes. The cultures were maintained in a humidified atmosphere with 10% CO_2_ in a Sanyo CO_2_ incubator (Osaka, Japan). The culture medium was renewed every 7th day and 1 day prior to an experiment. Confluent astrocytes cultures of an age between 14 and 28 days in culture were used for the experiments. Astrocyte-rich primary cultures are strongly enriched in astrocytes and contain only low amounts of contaminating microglial cells and oligodendrocytes [36, 37].

### Experimental Incubation of the Cells

For cell incubations, the medium was completely aspirated from the cultures, the cells were washed twice with 1 mL pre-warmed (37 °C) glucose-free incubation buffer (IB; 145 mM NaCl, 20 mM HEPES, 5.4 mM KCl, 1.8 mM CaCl_2_, 1 mM MgCl_2_, 0.8 mM Na_2_HPO_4_, pH adjusted with NaOH to 7.4 at 37 °C) and subsequently incubated for up to 5 h at 37 °C in the humidified atmosphere of a CO_2_-free incubator with 250 µL of IB that had been supplemented with pyruvate, other energy substrates, inhibitors of transporters and/or modulators of metabolic pathways. Appropriate solvent controls were performed for incubations with compounds that had been dissolved as concentrated stock solutions in DMSO. The final DMSO concentration in such media did not exceed 1.1% and the presence of DMSO in the concentrations used did not have any effect on the parameters investigated (data not shown). After the given incubation periods the incubation medium was harvested for determination of metabolite concentrations and cell viability. Cells were washed twice with 1 mL ice-cold (4 °C) phosphate-buffered saline (PBS; 10 mM potassium phosphate buffer pH 7.4 containing 150 mM NaCl) and either lysed as described below for ATP quantification or stored frozen until the protein determination was performed.

### Determination of Cell Viability and Initial Protein Content

To test for potential cell toxicity of a given treatment the extracellular activity of the cytosolic enzyme LDH was determined after the treatment for 10 µL media samples and compared with the initial cellular LDH activity of untreated cells, as previously described in detail [36]. Cellular protein content per well was determined by the Lowry method [38] using bovine serum albumin as standard protein.

### Determination of Extracellular Substrates and Metabolites

Pyruvate was quantified in a microtiter plate assay by the LDH and NADH-dependent reduction to lactate by a method adapted from Clarke and Payton [39]. Media volumes between 25 and 180 µL were diluted with 80 mM Tris-HCl buffer pH 7.2, containing 200 mM NaCl, to a total volume of 180 µL in wells of a microtiter plate before 180 µL reaction mixture (0.4 mM NADH and 4 U LDH in 80 mM Tris-HCl buffer pH 7.2, containing 200 mM NaCl) per well was added to reach a final initial NADH concentration of 0.2 mM. The decline observed in NADH absorbance determined at 340 nm after completion of the reaction (around 3 min) was measured in a microtiter plate spectrophotometer (Multiskan Sky microtiter spectrophotometer, Thermo Fisher Scientific, Schwerte, Germany) and used to calculate the pyruvate concentration in the sample.

Extracellular alanine was determined by a coupled enzymatic reaction in microtiter plates. In this assay, alanine is first transaminated by glutamate-pyruvate transaminase to pyruvate which is subsequently reduced by LDH to lactate and analyzed by the decrease in NADH absorbance at 340 nm. As the later reaction will also quantify pyruvate that may as well be present in the media samples analyzed, the concentration of alanine was calculated by the difference of signals obtained for reactions containing the complete reaction components and those containing only the pyruvate assay components. Media samples of 90 µL were mixed with 90 µL of the pyruvate reaction mixture (containing 0.8 mM NADH and 8 U LDH in 80 mM Tris-HCl buffer pH 7.2, containing 200 mM NaCl) in a microtiter plate well. After 3 min of incubation at room temperature (RT), the pyruvate-dependent decline in absorbance at 340 nm was completed and the concentration of pyruvate was calculated (pathlength of 0.5 cm for 180 µL per well). Thereafter, 180 µL of the second reaction mixture for alanine quantification (containing 0.7 U GPT and 10 mM α-ketoglutarate in 80 mM Tris-HCl buffer pH 7.2 containing 200 mM NaCl) was added. The decline in absorbance at 340 nm was measured after 90 min incubation at 37 °C in a humidified atmosphere. The concentration of pyruvate plus alanine was calculated (pathlength of 1 cm for 360 µL per well) from the decline in absorbance. The alanine concentration in the samples was calculated by subtracting the concentration of pyruvate determined (after addition of the first reaction mixture) from the sum of the concentrations of alanine plus pyruvate determined (after the addition of the second reaction mixture).

The concentration of extracellular glucose or lactate in the incubation medium was determined by coupled enzymatic assays as previously described in detail [36] for media sample volumes between 10 and 90 µL.

Extracellular acetate was quantified by using an acetate kit (Megazyme^©^ Acetic Acid Assay Kit, ACS Manual Format) according to the information provided by the supplier in a modification adapted to microtiter plates. The acetyl-coenzyme A synthetase (ACS) provided in the kit activates acetate to Acetyl-CoA which is subsequently combined with oxaloacetate to citrate (citrate synthase, CS). The oxaloacetate needed for this reaction is generated from malate by malate dehydrogenase (MDH) and this supply is quantified by the NADH-dependent increase in absorbance at 340 nm caused by the MDH reaction. For acetate quantification, 90 µL fresh media samples or acetate standards in incubation buffer (concentrations between 0 and 600 µM) were mixed with 90 µL of a first reaction mixture (containing CS and MDH) containing appropriate volumes of the kit solutions adapted to the microtiter plate format to determine acetate-independent reactions. After 4 min, 180 µL of the second reaction mixture of the kit (containing ACS) was added and the acetate-mediated increase in absorption at 340 nm was measured after 20 min of incubation at room temperature. Acetate concentrations in media samples were calculated by using the calibration curve obtained for the absorbances determined for the acetate standards.

Extracellular β-hydroxybutyrate (bHB) was determined according to Kientsch-Engel and Siess [40] using a modification that was adapted to microtiter plate formate. bHB is oxidised by bHB dehydrogenase (bHBDH) to acetoacetate and the NADH generated in this reaction is used to reduce the Fe^3+^-BPS complex to the Fe^2+^-BPS complex which strongly absorbs at 535 nm. For the assay, 180 µL of the bHB-containing incubation buffers or bHB standards (concentrations of up to 100 µM) were mixed in a well of a microtiter plate with 180 µL of a reaction mixture to obtain final concentrations of 350 mM potassium phosphate buffer pH 8.5, 8.8 mM NAD^+^, 0.45 mM FeCl_3_, 1.9 mM bathophenanthroline disulfonate (BPS), 3 µM phenazine methosulphate and 0.1 U bHBDH. The microtiter plate was then incubated for 90 min at room temperature in the dark before the absorbance of Fe^2+^-BPS at 535 nm was determined. bHB concentrations in media samples were calculated by using the calibration curve generated from the absorbances determined for the bHB standards.

### Quantification of Cellular ATP Content

Cellular ATP contents were determined for neutralized perchloric acid cell lysates of astrocyte cultures by using a commercial luciferase-based assay kit. After the indicated incubation, the media samples were collected and the cells were washed twice with 1 mL ice-cold PBS. Afterwards, the cells were lysed in 200 µL of ice-cold 0.5 M HClO_4_ on ice for 1 min. The cell lysates were collected and diluted by a factor of 20 in 0.5 M HClO_4_ before the pH was neutralised by the addition of an appropriate amount of 2 M KOH. Thereafter, the samples were vortexed and subsequently centrifuged for 5 min at 12,100×*g* to precipitate the KClO_4_ before the supernatant was transferred into a new cup. To adjust the pH, 10 µL of 1.4 M Tris-acetate buffer (pH 7.75) was added. ATP-standards in concentrations of up to 1000 nM in HClO_4_ were prepared and treated identically. Finally, 50 µL of each neutralised cell lysate or ATP standard was transferred into the wells of a black 96-well plate and mixed with 50 µL of the ATP detection reagent (Cell Titer Glo® 2.0 ATP Assay Kit). After 20 min of incubation in the dark at RT the luminescence signal was recorded by a Fluoroskan Ascent FL chemiluminescence plate reader (Thermo Fisher Scientific, Schwerte, Germany). ATP concentrations were calculated by comparison of the detected luminometric signals from the diluted cell lysates with the linear calibration curve of the values obtained from the ATP standards. Specific ATP contents were calculated by normalizing the determined ATP values per well to the initial cellular protein content per well.

### Staining for Mitochondrial Membrane Potential

After a given incubation period, cultured astrocytes were stained with tetramethylrhodamine ethyl ester (TMRE) to visualize the mitochondrial membrane potential [19]. The cells were incubated for 90 min in IB containing 0.5 mM pyruvate plus 40 nM TMRE in the absence or presence of 10 µM antimycin A or 1 µM BAM15. The incubation was performed at 37 °C in the humidified atmosphere of a cell incubator while the cell culture plates were wrapped in aluminum foil to prevent light exposure. After the incubation period, cellular fluorescence of TMRE was directly analyzed by fluorescence microscopy (Eclipse TE-2000-U with a DS-QiMc camera and imaging software NIS-Elements BR, Nikon, Düsseldorf, Germany) while the cells were kept in the incubation medium on a heating plate at 37 °C. The filter settings for detection of the fluorescence of TMRE were as follows: excitation at 510–565 nm, emission at 590 nm, dichromatic mirror at 575 nm. All images within one experiment were taken with the same light intensity and exposure time settings to allow for direct comparison of the different incubation conditions. TMRE fluorescence of the whole image section was quantified with the software ImageJ after subtraction of the background fluorescence using the rolling ball method within the software by applying the same radius for all images within one experiment.

### Presentation of Data and Statistical Analysis

The quantitative data shown are means ± SD of values obtained from three individual experiments performed in triplicates on independently prepared astrocyte cultures. Analysis for statistical significance of groups of data was performed by ANOVA followed by the Bonferroni post-hoc test using the software GraphPad InStat. The level of significance compared to control conditions are indicated by *p < 0.05, **p < 0.01 and ***p < 0.001. Analysis for statistical significance between pairs of data was calculated by the paired t-test. The level of significance between pairs is indicated by #p < 0.05, ##p < 0.01 and ###p < 0.001. p > 0.05 was considered as not significant.

## Results

### Consumption of Mitochondrial Substrates by Cultured Astrocytes

**Fig. 1:**
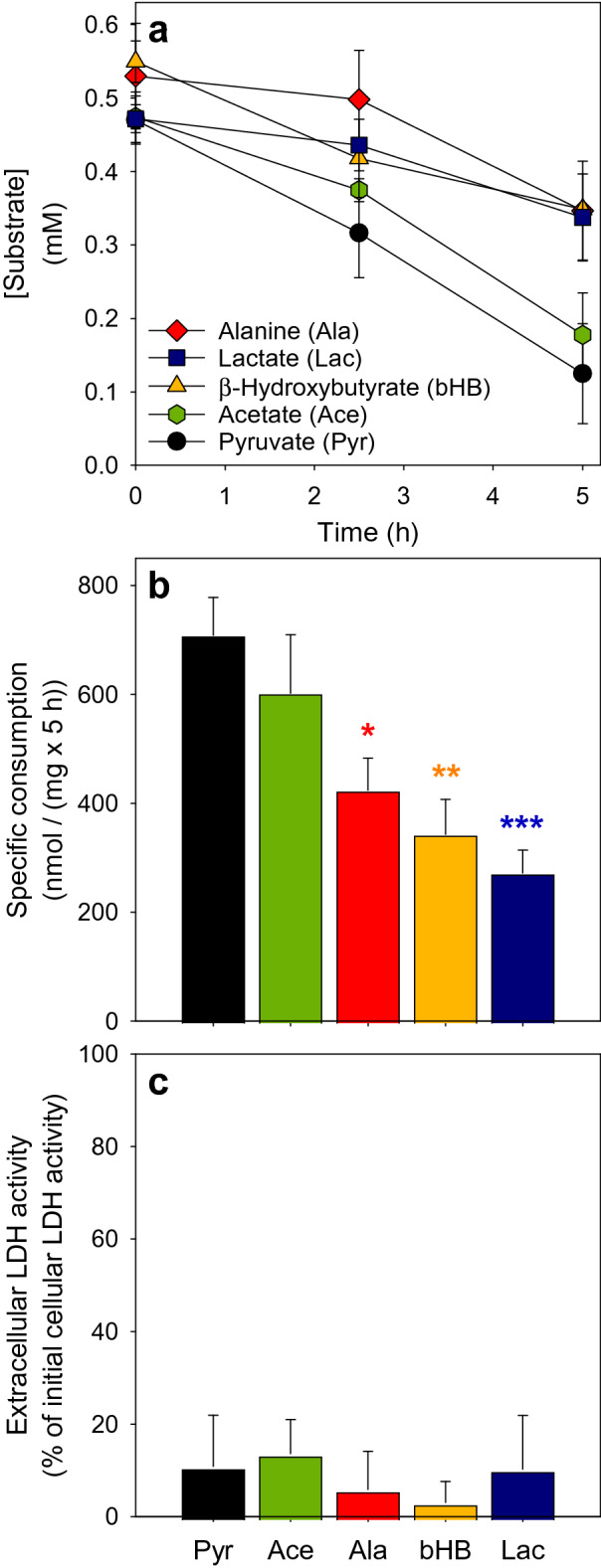
Consumption of mitochondrial substrates by primary astrocytes cultures. The cells were incubated in a glucose-free incubation buffer with 0.5 mM of the indicated substrates and the extracellular concentrations of the substrates applied were monitored over an incubation period of up to 5 h (**a**). In addition, the specific consumption of the applied substrates during the 5 h incubation (**b**) as well as the percental extracellular LDH activity (**c**) as indicator of a potential loss in cell viability were calculated. The data shown are means ± SD of values obtained in three experiments performed on independently prepared cultures. The initial cellular LDH activity of the cultures was 119 ± 14 nmol/(min × well) and the initial protein content was 126 ± 17 µg/well. In panel b, the significance of differences (ANOVA) compared to the values obtained for a pyruvate treatment is indicated by *p < 0.05, **p < 0.01 and ***p < 0.001

To test for the ability of astrocytes to take up and metabolize extracellular substrates that can be oxidized by mitochondrial metabolism, primary astrocyte cultures were exposed to 0.5 mM of pyruvate, lactate, acetate, alanine or bHB in a glucose-free incubation buffer and the decline in concentrations of the substances applied was recorded during an incubation period of up to 5 h (Fig. 1). All applied extracellular substrates disappeared at least partially from the incubation buffer during incubation of the cells, but the consumption of extracellular pyruvate was the highest among the tested substrates (Fig. 1a, b). The specific consumption rate of pyruvate was around 700 nmol/(mg × 5 h). A similar consumption rate was only observed for acetate, while the consumption rates calculated for incubations with alanine, bHB and lactate were significantly lower than those calculated for pyruvate (Fig. 1a, b). For comparison, cultured astrocytes that had been exposed to 0.5 mM glucose completely consumed the applied amount within the 5 h incubation period (data not shown). The viability of the cells was not affected by the treatments used, as indicated by the absence of any significant increase in extracellular LDH activity (Fig. 1c). In the absence of cells but otherwise under identical conditions, no decrease in the concentration of the applied substrates was observed (data not shown), demonstrating that viable cultured astrocytes can efficiently take up and metabolize the applied extracellular substrates.

### Concentration-Dependency of Pyruvate Consumption by Astrocytes

**Fig. 2:**
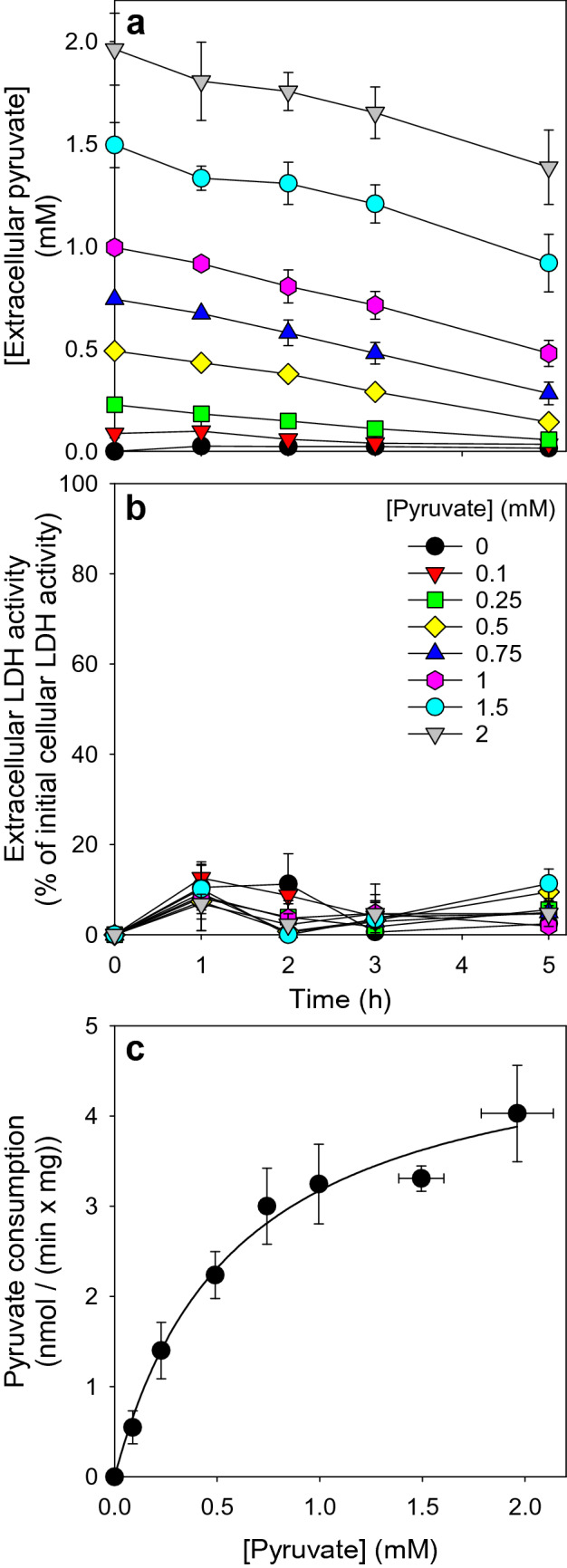
Time- and concentration-dependent consumption of extracellular pyruvate by primary astrocyte cultures. The cells were incubated for up to 5 h in a glucose-free incubation buffer with pyruvate in the concentrations indicated. The extracellular concentration of pyruvate (**a**) and the extracellular LDH activity (**b**) were determined for the time periods given. The almost linear decline in the extracellular pyruvate concentrations during the first 3 h of incubation was used to calculate the specific pyruvate consumption rates. The initial cellular LDH activity of the cultures was 116 ± 9 nmol/(min × well) and the initial protein content was 122 ± 7 µg/well (**c**). Half-maximal pyruvate consumption (as calculated by using the Michaelis-Menten equation) was observed for an initial pyruvate concentration of 0.6 ± 0.1 mM and the maximal pyruvate consumption rate was calculated to be 5.1 ± 0.8 nmol/(min × mg). The data shown are means ± SD of values obtained in three experiments performed on independently prepared cultures

To test for the concentration dependency of pyruvate consumption, cultured astrocytes were exposed to different concentrations of pyruvate and the loss in extracellular pyruvate was monitored (Fig. 2). For all concentrations applied, the extracellular pyruvate levels declined almost proportional with time (Fig. 2a) and the viability of the cells was not compromised as indicated by the absence of any significant increase in extracellular LDH activity (Fig. 2b). Calculation of the pyruvate consumption rates per minute for the initial 3 h of incubation revealed a hyperbolic relationship between the specific consumption rate and the concentration of pyruvate initially applied (Fig. 2c). Analysis of the data obtained by the Michaelis-Menten equation revealed half-maximal pyruvate consumption for an initial pyruvate concentration of 0.6 ± 0.1 mM and a maximal pyruvate consumption rate of 5.1 ± 0.8 nmol/(min × mg).

### Inhibition of Astrocytic Pyruvate Consumption by an MCT1 Inhibitor or MCT1 Substrates

**Fig. 3:**
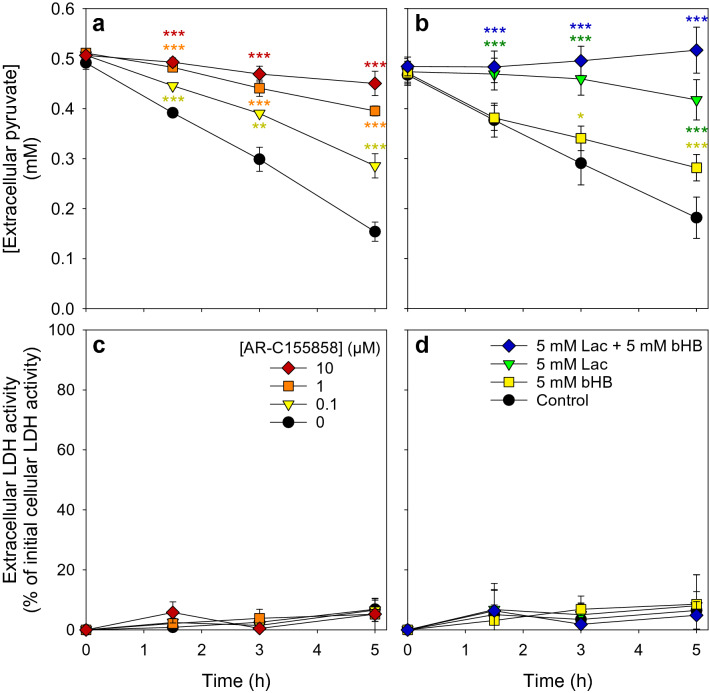
Modulation of astrocytic pyruvate consumption by an MCT1 inhibitor or MCT1 substrates. Astrocyte cultures were incubated for up to 5 h in a glucose-free incubation buffer with 0.5 mM pyruvate in the absence or the presence of the MCT1 inhibitor AR-C155858 in the concentrations indicated (**a**, **c**) or in the absence or the presence of 5 mM of the MCT1 substrates lactate (Lac) and/or beta-hydroxybutyrate (bHB; **b**, **d**). The extracellular concentration of pyruvate (**a**, **b**) and the extracellular LDH activity (**c**, **d**) were determined for the incubation times indicated. The initial cellular LDH activities of the cultures were 150 ± 26 nmol/(min × well) (**a**, **c**) and 138 ± 18 nmol/(min × well) (**b**, **d**). The initial protein contents of the cultures were 113 ± 2 µg/well (**a**, **c**) and 129 ± 8 µg/well (**b**, **d**). The data shown are means ± SD of values obtained in three experiments performed on independently prepared cultures. The significance of differences (ANOVA) compared to the values obtained for the control incubation (no inhibitor or no other MCT1 substrate) is indicated by *p < 0.05 and ***p < 0.001

Pyruvate is considered to be transported into astrocytes via MCT1 [32, 33, 41]. To test whether MCT1-mediated uptake is involved in the observed pyruvate consumption by astrocytes, we studied the consequences of an application of the MCT1 inhibitor AR-C155858 [42–44] or of the known MCT1 substrates lactate and bHB [32, 35, 45] on the consumption of 0.5 mM pyruvate. Application of AR-C155858 caused a concentration-dependent impairment of the pyruvate consumption and 10 µM of the inhibitor lowered the pyruvate consumption by around 80% (Fig. 3a). In addition, a 10 times excess of lactate or bHB significantly lowered the pyruvate consumption, while the consumption was completely prevented in the presence of both lactate plus bHB (Fig. 3b). None of the conditions applied caused any obvious cell toxicity as indicated by the absence of any increase in extracellular LDH activity (Fig. 3c, d).

### Pyruvate Consumption in the Presence of Lactate and Glucose

**Fig. 4:**
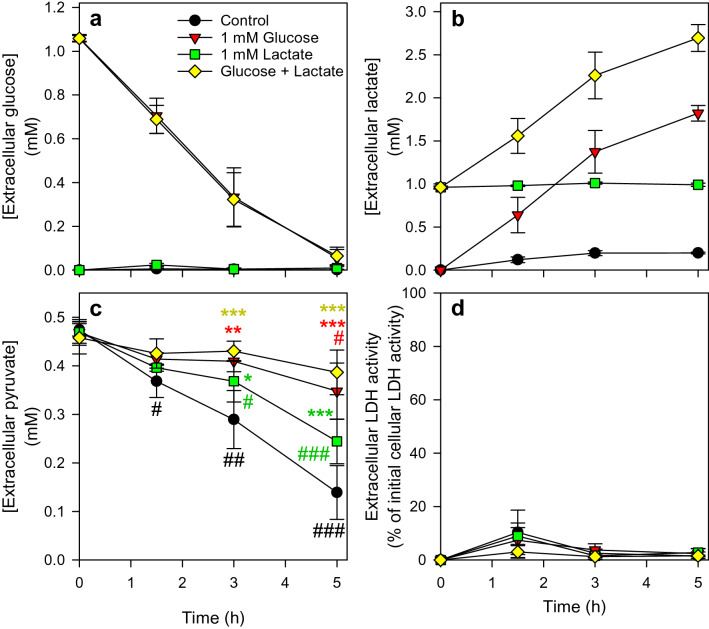
Pyruvate consumption by cultured astrocytes in the presence of glucose and/or lactate. Cultured primary astrocytes were incubated for up to 5 h in a glucose-free incubation buffer with 0.5 mM pyruvate in the absence or the presence of 1 mM glucose and/or 1 mM lactate. The extracellular concentrations of glucose (**a**), lactate (**b**), pyruvate (**c**) and the extracellular LDH activity (**d**), as indicator of a potential loss in cell viability, were monitored over an incubation period of up to 5 h. The initial cellular LDH activity of the cultures was 150 ± 38 nmol/(min × well) and the initial protein content was 124 ± 21 µg/well. The data shown are means ± SD of values obtained in three experiments performed on independently prepared cultures. In panel c, the significance of differences (ANOVA) compared to the values obtained for the control incubation (only pyruvate) is indicated by *p < 0.05, **p < 0.01 and ***p < 0.001 and the significance of differences (ANOVA) compared to the values obtained for the respective initial pyruvate concentrations is indicated by #p < 0.05, ##p < 0.01 and ###p < 0.001

An excess of lactate (5 mM) lowered pyruvate consumption by astrocytes (Fig. 3b). To test whether the presence of lower concentrations of lactate as well as of lactate formed during incubation of astrocytes with glucose will affect pyruvate consumption, astrocytes were exposed to 0.5 mM pyruvate in the absence or the presence of 1 mM lactate and/or 1 mM glucose. After application of 1 mM glucose, almost all the glucose was consumed during incubation of the cells for 5 h (Fig. 4a) and around 1.7 mM of lactate were found released from the cells (Fig. 4b). Also for such conditions pyruvate was found to be consumed by the cells, although this consumption was significantly lowered by the presence of lactate and/or glucose (Fig. 4c). None of the conditions applied caused any obvious cell toxicity as indicated by the absence of any increase in extracellular LDH activity (Fig. 4d).

### Pyruvate Consumption in the Presence of an Inhibitor of the Mitochondrial Pyruvate Carrier

**Fig. 5:**
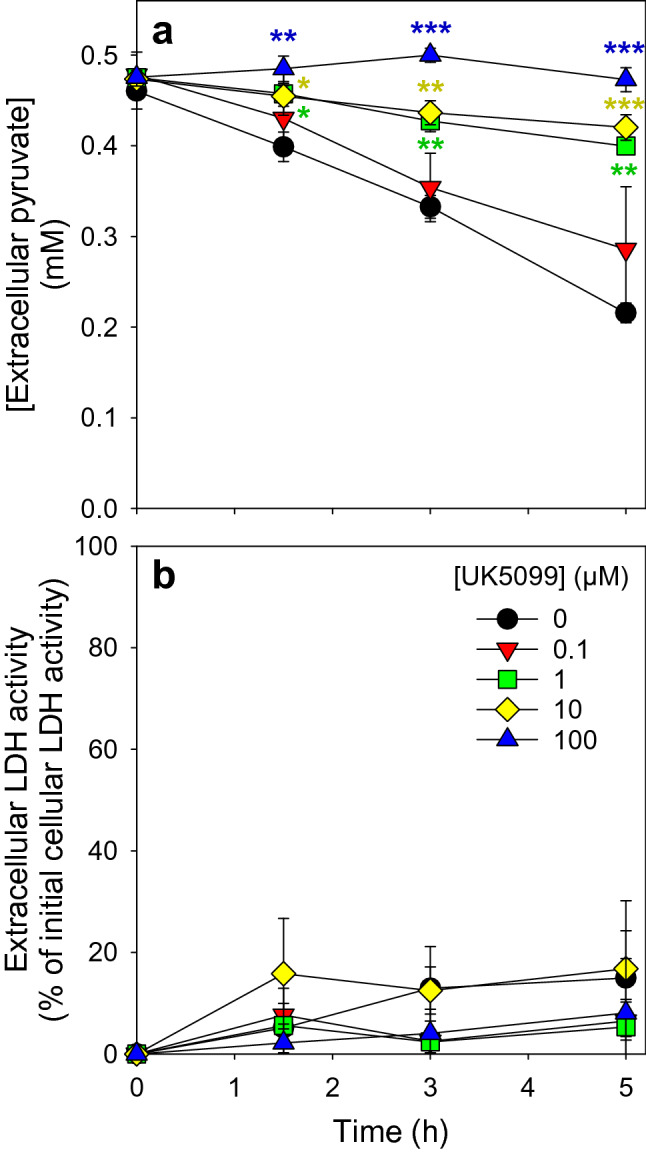
Modulation of astrocytic pyruvate consumption by inhibition of the mitochondrial pyruvate carrier. Cultured astrocytes were incubated for up to 5 h in a glucose-free incubation buffer with 0.5 mM pyruvate in the absence or the presence of UK5099, an inhibitor of the mitochondrial pyruvate carrier, in the concentrations indicated. The extracellular concentration of pyruvate (**a**) and the extracellular LDH activity (**b**), as indicator of a potential loss in cell viability, were determined for the indicated time points of incubation. The initial cellular LDH activity of the cultures was 93 ± 6 nmol/(min × well) and the initial protein content of the cultures was 104 ± 10 µg/well. The data shown are means ± SD of values obtained in three experiments performed on independently prepared cultures. The significance of differences (ANOVA) compared to the values obtained for the control incubation (no inhibitor) is indicated by *p < 0.05, **p < 0.01 and ***p < 0.001

Mitochondrial uptake is a prerequisite of a subsequent mitochondrial metabolism of pyruvate. To test whether the mitochondrial pyruvate carrier (MPC) is involved in the observed astrocytic pyruvate consumption, we applied UK5099, an inhibitor of the mitochondrial pyruvate carrier [23, 46, 47]. The presence of UK5099 lowered the astrocytic pyruvate consumption in a concentration-dependent manner and completely prevented pyruvate consumption in a concentration of 100 µM (Fig. 5a). The viability of the cells was not affected by the presence of the inhibitor as indicated by the absence of any significant increase in extracellular LDH activity (Fig. 5b).

### Consequences of a Modulation of Mitochondrial Metabolism on Astrocytic Pyruvate Consumption

**Fig. 6:**
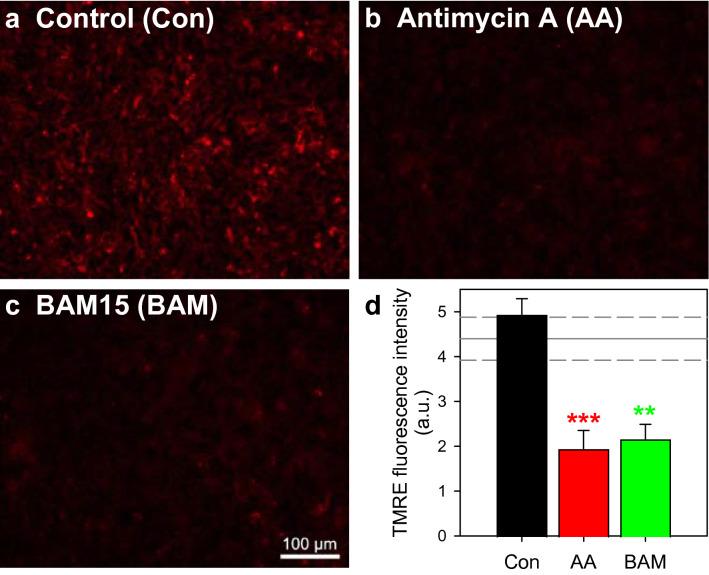
Consequences of an application of modulators of mitochondrial metabolism on the mitochondrial membrane potential of cultured astrocytes. The cultures were incubated for 90 min without (**a**, **d**) or with 10 µM of the complex III inhibitor antimycin A (**b**, **d**) or 1 µM of the uncoupler BAM15 (**c**, **d**) before the cells were stained with TMRE to indicate the intensity of the mitochondrial membrane potential. Panels a to c show pictures of stained cultures from a representative experiment. The scale bar in panel c represents 100 μm and applies to panels a-c. Panel d shows the quantification of the mitochondrial TMRE staining for the conditions applied and the data presented are means ± SD obtained from three experiments performed on independently prepared cultures. The cellular TMRE fluorescence of glucose-fed cultures was 4.4 ± 0.5 a.u. and is indicated in panel d as horizontal lines. In panels d, the significance of differences (ANOVA) compared to the data for control incubations is indicated by **p < 0.01 and ***p < 0.001

**Fig. 7:**
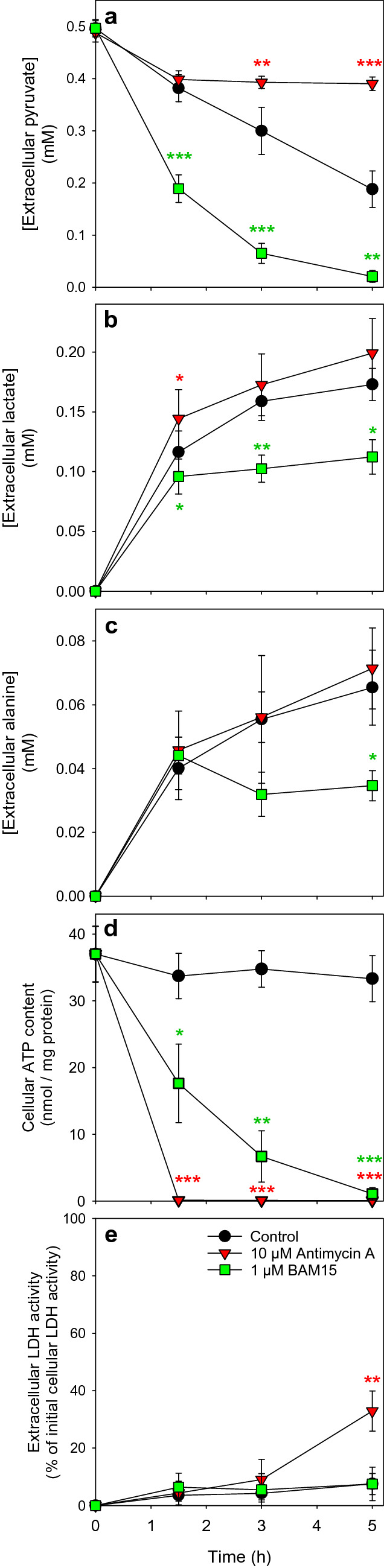
Pyruvate consumption by astrocytes in the presence of antimycin A or BAM15. Primary astrocyte cultures were incubated for up to 5 h with 0.5 mM pyruvate in the absence or the presence of either 10 µM of the mitochondrial complex III inhibitor antimycin A or 1 µM of the uncoupler BAM15 before the extracellular concentrations of pyruvate (**a**), lactate (**b**), alanine (**c**), the specific cellular ATP content (**d**) and the extracellular LDH activity (**e**) were determined. The initial cellular protein content was 108 ± 12 µg/well and the initial cellular LDH activity 120 ± 16 nmol/(min × well). The data shown are means ± SD obtained from three experiments performed on independently prepared cultures. The significance of differences (ANOVA) compared to the data for controls without antimycin A and BAM15 are indicated by *p < 0.05, **p < 0.01 and ***p < 0.001

**Fig. 8:**
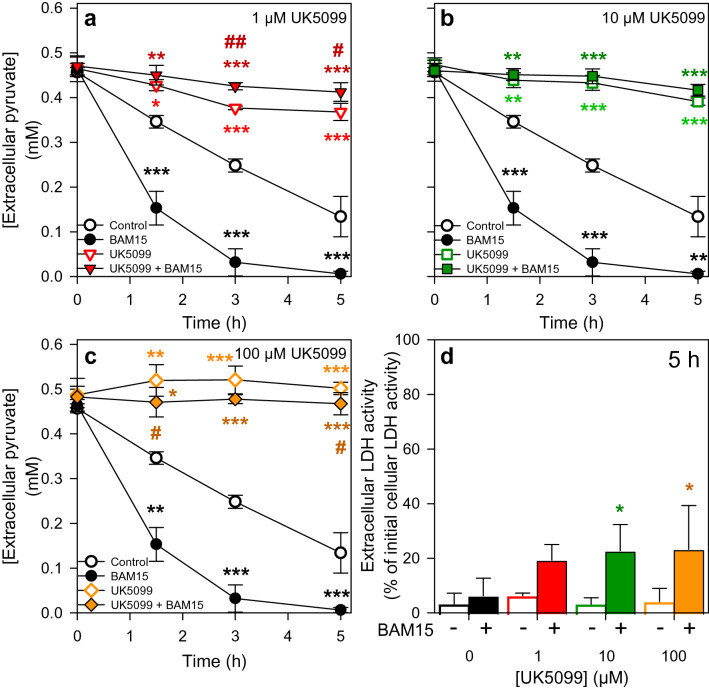
Pyruvate consumption in astrocytes in the presence of an inhibitor of the mitochondrial pyruvate carrier and/or the uncoupler BAM15. Cultured primary astrocytes were incubated for up to 5 h in a glucose-free incubation buffer with 0.5 mM pyruvate in the absence or the presence of 1 µM (**a**), 10 µM (**b**) or 100 µM (**c**) UK5099, an inhibitor of the mitochondrial pyruvate carrier and/or 1 µM of the uncoupler BAM15. The extracellular concentrations of pyruvate (**a**, **b**, **c**) and the extracellular LDH activity (**d**), as indicator of a potential loss in cell viability, were determined for the indicated incubation periods. The initial cellular LDH activity of the cultures was 167 ± 26 nmol/(min × well) and the initial protein content of the cultures was 134 ± 21 µg/well. The data shown are means ± SD of values obtained in three experiments performed on independently prepared cultures. The significance of differences (ANOVA) compared to the values obtained for the control incubation (no inhibitor) is indicated by *p < 0.05, **p < 0.01 and ***p < 0.001. The significance of differences (t-test) between incubations containing UK5099 and UK5099 plus BAM15 is indicated by #p < 0.05 and ##p < 0.01

To investigate to which extend mitochondrial oxidation may be involved in the observed pyruvate consumption, we incubated cultured astrocytes with 0.5 mM pyruvate in the presence of substances that are known to interfere with mitochondrial metabolism, such as the complex III inhibitor antimycin A [48, 49] and the respiratory chain uncoupler BAM15 [50]. Exposure of cultured astrocytes for 90 min to those substances lowered the mitochondrial membrane potential significantly (Fig. 6), but did not cause acute toxicity as indicated by the absence of any rapid increase in extracellular LDH activity (Fig. 7e). Pyruvate-treated cells consumed the applied pyruvate almost proportional to the time (Fig. 7a), maintained the high initial ATP content throughout an incubation for up to 5 h (Fig. 7d) and remained viable during this incubation (Fig. 7e). In contrast, pyruvate consumption in antimycin A-treated astrocytes was abolished following the initial incubation period of 90 min. Those cells contained already after 90 min hardly any ATP (Fig. 7d) and the viability of the cells was compromised as demonstrated by the significant increase in extracellular LDH activity found after 5 h of incubation (Fig. 7e). For BAM15-treated astrocyte cultures an accelerated pyruvate consumption was observed (Fig. 7a) that was accompanied by a gradual decline of cellular ATP contents throughout the 5 h incubation (Fig. 7d), while the cell viability was not compromised under those conditions (Fig. 7e). The strong acceleration of pyruvate consumption in BAM15-treated astrocytes (Figs. 7a and 8a-c) was prevented, in addition to the basal pyruvate consumption, in the presence of the MPC inhibitor UK5099 in concentrations as low as 1 µM (Fig. 8a–c). Such coincubations of astrocyte cultures with BAM15 and UK5099 caused some cell toxicity as demonstrated by the significant increase in extracellular LDH activity after 5 h of incubation (Fig. 8d).

### Test for Release of Lactate and Alanine as Potential Products of Pyruvate Metabolism

As pyruvate has been reported to be metabolized by astrocytes to lactate and alanine [31], the extracellular concentrations of lactate and alanine were determined for cultures that had been exposed in the absence or the presence of 0.5 mM pyruvate (Fig. 7b, c). After a 3 h incubation in the absence of pyruvate, 34 ± 9 µM lactate and 32 ± 3 µM alanine were determined extracellularly, while higher levels of 159 ± 16 µM lactate and 55 ± 20 µM alanine were quantified for cells that had been exposed for 3 h to 0.5 mM pyruvate (Table 1). In contrast to those metabolites, no pyruvate-derived extracellular acetate and bHB were detected in astrocytes cultures that had been incubated with or without pyruvate (data not shown). The concentrations of extracellular lactate and alanine that were found for astrocytes incubated without pyruvate were subtracted from the respective concentrations of lactate or alanine that had been released from pyruvate-treated cells to quantify the pyruvate-derived lactate and alanine (Table 1). Within 3 h of incubation the cells consumed 481 ± 34 nmol/mg pyruvate and released 289 ± 17 nmol/mg pyruvate-derived lactate and 52 ± 36 nmol/mg pyruvate-derived alanine. Thus, extracellular lactate and alanine accounted for around 60% and 10%, respectively, of the pyruvate that had been consumed by the pyruvate-exposed cells (Table 1). Antimycin A-treated astrocytes consumed less pyruvate than control cells but similar concentrations of extracellular lactate and alanine were found, while extracellular lactate and alanine concentrations were significantly lower in BAM15-treated astrocytes (Fig. 7b, c), despite of the accelerated pyruvate consumption (Fig. 7a).

**Table 1:** Formation of lactate and alanine from the pyruvate consumed by astrocytes

	(µM)	(nmol/mg)	(%)
Pyruvate consumed			
0.5 mM Pyruvate	208 ± 28	481 ± 34	100.0 ± 7.1
Lactate released			
0.5 mM Pyruvate	159 ± 16	367 ± 7	
0 mM Pyruvate	34 ± 9	77 ± 12	
Δ (0.5–0 mM)	125 ± 8	289 ± 17	60.3 ± 4.1
Alanine released			
0.5 mM Pyruvate	55 ± 20	126 ± 34	
0 mM Pyruvate	32 ± 3	73 ± 3	
Δ (0.5–0 mM)	24 ± 17	52 ± 36	10.9 ± 7.9

Primary astrocyte cultures were incubated for 3 h without (0 mM) or with 0.5 mM pyruvate before the extracellular concentrations of pyruvate, lactate and alanine were determined. Pyruvate consumption as well as lactate or alanine release are given for the conditions investigated as µM and nmol/mg protein. In addition, for lactate and alanine release the difference between treatments with and without pyruvate (Δ 0.5-0 mM) were calculated and are given as µM, nmol/mg and as percent of the pyruvate consumed. The data shown are means ± SD obtained from three experiments performed on independently prepared cultures. The initial cellular protein content was 108 ± 12 µg/well

## Discussion

Pyruvate is an important metabolite that links cytosolic glycolysis with mitochondrial metabolism [6, 7]. As pyruvate can also be released and taken up by astrocytes [29, 31, 51], we have investigated the astrocytic metabolism of extracellular pyruvate by using astrocyte primary cultures as a model system. Here we report that astrocytes efficiently consume and metabolize pyruvate and that its consumption strongly depends on defined transport processes and mitochondrial activity.

Astrocytes are able to take up glucose efficiently and are considered to convert the internalized glucose via glycolysis mainly to lactate with quantitatively less mitochondrial pyruvate oxidation [2]. However, in addition to glucose, a variety of other extracellular substrates, including lactate, pyruvate, alanine, bHB and acetate, have been reported to be metabolized by astrocytes, at least in culture, and can be used as source for energy production and/or synthesis of other metabolites [4, 13, 14, 52]. In our study, these literature data on the consumption of the listed monocarboxylates were confirmed for cultured primary rat astrocytes.

The transporter primarily responsible for the uptake of the monocarboxylates pyruvate, lactate, bHB and acetate into astrocytes is MCT1 [32, 41, 53], while alanine is taken up into astrocytes mainly via the system L isoform LAT2 and partially via ASCT2 [54]. When comparing the different MCT1 substrates, pyruvate and acetate were found to be consumed more efficiently by astrocytes than bHB and lactate. Reasons for the observed difference in consumption rates are the low concentration of substrates applied (0.5 mM) and the different kinetic parameters of MCT1 for the substrates investigated. Reported K_m_ values for MCT1-mediated uptake are low for pyruvate (1 mM; [35]) and acetate (1.6 mM; [55]), but much higher for lactate (3.5–10 mM; [32, 35, 56]) and bHB (12.5 mM; [57]). Using these K_m_ values to calculate initial transport velocities by the Michaelis-Menten equation for a substrate concentration of 0.5 mM revealed that compared to the uptake velocity for pyruvate (100%) the initial uptake velocities of the other MCT1 substrates were lower with 71% for acetate, 14–38% for lactate and 11% for bHB. This explains why uptake and consumption of pyruvate and acetate by astrocytes are favored at a concentration of 0.5 mM in comparison to lactate and bHB.

The consumption of pyruvate by cultured astrocytes depends strongly on the initial pyruvate concentration applied. A half-maximal consumption rate was calculated for an initial pyruvate concentration of 0.6 ± 0.1 mM and the maximal specific consumption rate was found to be 5.1 ± 0.8 nmol/(min × mg). These kinetic data for pyruvate consumption for an incubation period of 3 h fit well with data reported for the initial (5 min) uptake of ^14^ C-labelled pyruvate revealing a K_m_ value of 1.0 ± 0.3 mM and a V_max_ value of 7.5 ± 0.4 nmol/(min × mg) for cellular pyruvate accumulation in primary astrocytes [31]. The half-maximal consumption rate determined for pyruvate (0.6 mM) is also similar to the reported K_m_ value for pyruvate uptake (1.01 ± 0.06 mM) in MCT1-expressing *Xenopus laevis* oocytes [35]. The contribution of MCT1 as the plasma membrane transporter responsible for the observed astrocytic pyruvate consumption is strongly supported by the impairment of pyruvate consumption in the presence of the MCT1 inhibitor AR-C155858 [42–44] or of an excess of the competing MCT1 substrates lactate and bHB [32, 41–44]. All these data are consistent with the view that MCT1 is mainly responsible for pyruvate uptake in astrocytes [33, 41, 56] and demonstrate that MCT1-mediated pyruvate uptake is a prerequisite for pyruvate consumption by astrocytes. Although MCT1 seems to play the main role in the uptake of pyruvate, a small part of pyruvate consumption could not be blocked by application of the MCT1 and 2 inhibitor AR-C155858. This could be due to the presence of the monocarboxylate transporter MCT4 which has been reported to be expressed in primary rat astrocyte cultures [58]. This transporter was originally thought to have a rather high K_M_-value for pyruvate of around 36 mM [58], but a more recent study suggests a lower value of around 4 mM for pyruvate transport by MCT4 [59]. Thus, MCT4 could at least partially contribute to the astrocytic pyruvate consumption that is  insensitive to MCT1-inhibition.

Pyruvate oxidation to CO_2_ is a mitochondrial process that requires uptake of pyruvate through the inner mitochondrial membrane. This transport process is mediated by the mitochondrial pyruvate carrier (MPC) [22]. Mitochondrial uptake and metabolism of pyruvate appear to be key components of the observed pyruvate consumption by astrocytes as the presence of UK5099, an inhibitor of MPC [23, 46], almost completely abolished pyruvate consumption. Similarly, inhibition of mitochondrial respiration by antimycin A impaired the consumption of extracellular pyruvate by astrocytes as the NAD^+^ consumed by pyruvate dehydrogenase and by the citric acid cycle during the complete oxidation of pyruvate to CO_2_ cannot be regenerated by an inhibited respiratory chain [48, 60]. In contrast, BAM15-mediated uncoupling of the respiratory chain from mitochondrial ATP synthesis [50], increased pyruvate consumption, most likely by the accelerated mitochondrial NADH oxidation and NAD^+^ regeneration for further mitochondrial pyruvate oxidation to CO_2_. This is consistent with accelerated oxygen consumption in BAM15-treated C2C12 mouse myotubes [61] and BAM15-treated mouse liver mitochondria [50]. Finally, the accelerated (and the normal) pyruvate consumption in BAM15-treated astrocytes was abolished by inhibition of the MPC and some toxicity was observed for this condition, demonstrating that mitochondrial pyruvate uptake is required for the BAM15-induced accelerated pyruvate consumption and for maintaining cell viability under such conditions. Already at the low concentration of 1 µM UK5099 was sufficient to prevent the BAM15-induced mitochondrial pyruvate consumption in cultured astrocytes, showing that indeed inhibition of MPC is the reason for the observed impairment of astrocytic pyruvate consumption by UK5099. For applications of high concentration of UK5099 such as 100 µM a potential partial contribution of an inhibition of MCTs by UK5099 cannot be excluded as UK5099 has been reported to also have some inhibitory potential on MCTs [62], although the Ki value of UK5099 for MCT-mediated transport is two to three orders of magnitude higher than that for MPC [57, 63, 64]. All these data strongly underline the importance of astrocytic MPC and functional oxidative phosphorylation for the mitochondrial metabolism of pyruvate in astrocytes.

Astrocytes that had been incubated with pyruvate in the absence of glucose maintained their viability, their mitochondrial membrane potential and a high cellular ATP content. In contrast, both antimycin A and BAM15 lowered the mitochondrial membrane potential to a similar extend and depleted the cells of ATP in the presence of pyruvate. However, application of antimycin A depleted the cells almost completely of ATP already within 1.5 h, while a gradual loss in cellular ATP content was observed for BAM15-treated cells. Consistently, cell toxicity was found for antimycin A-treated and pyruvate exposed astrocytes, but not for the respective BAM15-treated cells. These data demonstrate that mitochondrial ATP production is essential to maintain a high ATP content in pyruvate-treated astrocytes. Furthermore, these findings suggest that in BAM15-treated cells a residual capacity of the respiratory chain is available to at least partially slow down ATP loss in the uncoupled situation, in contrast to the antimycin A treatment that completely inhibited the electron flow through the respiratory chain.

Substantial amounts of extracellular lactate and alanine were determined in glucose-deprived and pyruvate-treated astrocytes. Part of the extracellular lactate and alanine may be derived from residual free glucose, glycolysis intermediates and/or glycogen that is mobilized after glucose deprivation [19, 65, 66]. However, even after correction of the values obtained from pyruvate-treated astrocytes for the respective data from pyruvate-free control incubations, the amounts of lactate and alanine determined accounted to around 60% and 10%, respectively, of the total amount of pyruvate consumed by the cells. Thus, the pyruvate consumed by astrocytes within 3 h appears to have been metabolized mainly to lactate and to a lower extent to alanine, both of which had been exported from the cells. The excessive metabolism of pyruvate to lactate is consistent with the reported rapid and substantial metabolism within minutes of internalized ^14^ C-pyruvate to ^14^ C-lactate in cultured astrocytes [31] and with the loss of cellular lactate from astrocytes in vivo after injection of a pyruvate-containing solution [67].

The low amounts of pyruvate-derived alanine are most likely generated by glutamate-pyruvate transaminase [18, 21] via transamination of internalized pyruvate with amino groups that are derived from amino acids present at the onset of the incubation. In contrast, the electrons required for pyruvate reduction to lactate via LDH are unlikely to be only derived from initially present cytosolic NADH. The cellular NADH pool in cultured astrocytes accounts for only 0.70 ± 0.03 nmol/mg [68] and, even if the entire amount would be present as cytosolic NADH, it would have to be recycled more than 400 times by cytosolic processes to allow the formation of the pyruvate-derived lactate that was found extracellularly (around 300 nmol/mg). This appears highly unlikely for glucose-depleted astrocytes, as they are unable to sustain their NADH levels under glucose-depletion [19, 68]. It appears more likely that the large amounts of NADH needed for cytosolic pyruvate reduction under the conditions studied are derived from mitochondria, where NADH is continuously produced by mitochondrial pyruvate oxidation via the PDH and the citric acid cycle dehydrogenases.

Lactate and alanine account for around 70% of the pyruvate consumed. Assuming that the residual 30% of consumed pyruvate (around 145 nmol/mg) would have been fully oxidized in mitochondria to CO_2_ to yield 4 NADH per pyruvate oxidized [7], a total amount of 580 nmol/mg NADH would have been generated by mitochondrial PDH and citric acid cycle. This would account for almost twice the amount needed for the generation of the pyruvate-derived lactate that was found to be released into the medium. However, mitochondrial NADH cannot be used directly for cytosolic LDH-dependent reduction of pyruvate and the electrons need to be metabolically transported [18]. A shuttle that could contribute to the electron transfer from mitochondrial NADH via the inner mitochondrial membrane to generate cytosolic NADH is the bidirectional malate-aspartate shuttle [69, 70]. Such a transfer of reducing equivalents from mitochondria to cytosol has already been postulated for rat liver cells [71] and is also required for the transfer of oxaloacetate as malate from the mitochondrial matrix to the cytosol to provide substrate for astrocytic glyconeogenesis [72, 73]. Furthermore, the higher levels of lactate found for antimycin A-treated, pyruvate-exposed astrocytes would be consistent with a potential use of mitochondrial NADH for pyruvate reduction to lactate. As efficient NADH oxidation via the respiratory chain is impaired by the antimycin A treatment [48, 60], more mitochondrial NADH would be available to supply electrons for malate-aspartate shuttle-mediated transport into the cytosol. Conversely, the NADH produced in BAM15-treated astrocytes during rapid mitochondrial oxidation of pyruvate is likely to be instantly oxidized by the uncoupled respiratory chain, thereby lowering the potential of mitochondrial NADH to feed electrons into the malate-aspartate shuttle. Further studies are now required to experimentally elucidate to which extent electrons derived from pyruvate oxidation are shuttled from the mitochondrial matrix into the cytosol to support cytosolic pyruvate reduction to lactate.

Extracellular pyruvate has been reported to be present in brain in concentrations of around 160 µM [74, 75] and pyruvate concentrations in the cerebrospinal fluid range between 30 and 200 µM [76–78]. Thus, the observed consumption of micromolar concentrations of extracellular pyruvate by cultured astrocytes appears to take place at extracellular pyruvate concentrations that are physiologically present in brain. By their ability to release pyruvate [20, 29] and to consume extracellular pyruvate [31] (present report), astrocytes may regulate extracellular pyruvate levels to establish a suitable concentration that is sufficiently high to allow the reported neuroprotective function of astrocyte-derived extracellular pyruvate [30, 79, 80].

In conclusion, the results presented here demonstrate that in glucose-deprived astrocytes the pyruvate transporters MCT1 and MPC are mainly mediating the cellular uptake of pyruvate into astrocytes and the transport of pyruvate from cytosol into mitochondria, respectively. In addition, mitochondrial activity appears to be the main regulator for the consumption of extracellular pyruvate. The ultimate end-point of pyruvate oxidation will be CO_2_, but we assume that the fate of extracellular pyruvate that is taken up by astrocytes will strongly differ depending on the metabolic situation of the cells and the respective need for potential products of pyruvate metabolism, such as alanine, acetyl-CoA, oxaloacetate or intermediates of the citric acid cycle. Further studies are now required to explore in more detail the generation of pyruvate-derived products in cells and media of cultured astrocytes for various metabolic conditions. For such studies the mass spectroscopic analysis of metabolic products derived from ^13^ C-labeled pyruvate should be considered as a suitable approach as previously demonstrated for other studies on astrocytic metabolism [81, 82].

## Data Availability

Enquiries about data availability should be directed to the authors.
